# Prediction of child nutritional status using deep neural networks: A cross-sectional study of Egypt DHS data (2005–2014)

**DOI:** 10.1097/MD.0000000000046089

**Published:** 2025-11-21

**Authors:** Abdelaziz Hendy, Rasha Kadri Ibrahim, Sally Mohammed Farghaly Abdelaliem, Hosny Maher Sultan, Shaban Majed Sinnokrot, Mohammad Al-Ma’ani, Badriah Alzahrani, Waad Hasan Ali, Afrah Madyan Alshammari, Ahmed Hendy

**Affiliations:** a Department of Maternal and Child Health, College of Nursing, Qassim University, Buraydah, Saudi Arabia; b Nursing Department, Fatima College of Health Sciences, Al Dhafra region, Madinat Zayed, UAE; c Department of Nursing Management and Education, College of Nursing, Princess Nourah bint Abdulrahman University, Riyadh, Saudi Arabia; d Nursing Department, College of Pharmacy and Health Sciences, Ajman University, Ajman, UAE; e Department of Clinical Nursing, Faculty of Nursing, Zarqa University, Zarqa, Jordan; f Faculty of Nursing, Philadelphia University, Amman, Jordan; g Department of Social Sciences, College of Arts and Humanities, Jazan University, Jazan, Saudi Arabia; h Department of Nursing Management and Education, College of Nursing, Princess Nourah bint Abdulrahman University, Riyadh, Saudi Arabia; i Department of Maternal and Child Health Nursing, College of Nursing, Jouf University, Sakaka, Saudi Arabia; j Department of Computational Mathematics and Computer Science, Institute of Natural Sciences and Mathematics, Ural Federal University, Yekaterinburg, Russian Federation; k Department of Mechanics and Mathematics, Western Caspian University, Baku, Azerbaijan.

**Keywords:** child nutrition, deep neural network, health monitoring, machine learning, wasting

## Abstract

Child malnutrition, particularly wasting and undernutrition, remains a pressing public health issue in low- and middle-income countries. Timely prediction of nutritional status using accessible demographic and health indicators can support early interventions. This study explores the use of a deep neural network (DNN) model to predict child nutritional status using nationally representative survey data from Egypt. To develop and evaluate the performance of a DNN model for classifying child nutritional status and to compare its performance against traditional machine learning models, including decision tree and Random Forest classifiers. This is a retrospective cross-sectional study based on pooled data from the Egypt Demographic and Health Surveys conducted in 2005, 2008, and 2014. A total of 36,313 children under the age of 5 with complete anthropometric and demographic data were included. A deep neural network was trained on the Egypt Demographic and Health Surveys dataset using class weighting and synthetic minority oversampling technique to address class imbalance. Performance was assessed using accuracy, precision, recall, harmonic mean of precision and recall (F1-score), and receiver operating characteristic (ROC)–area under the curve (AUC) and compared against decision tree and random forest classifiers. The DNN outperformed all baseline models, achieving an accuracy of 89%, recall of 91%, and an ROC–AUC of 0.95. SHAP analysis revealed that maternal body mass index, child age, birth weight, and household wealth index were the most influential features. The random forest achieved a ROC–AUC of 0.95 but showed lower recall and F1-scores compared to the DNN. The DNN model demonstrated high performance in predicting child nutritional status and shows promise as a public health screening tool. Future work should focus on external validation across different populations and enhanced interpretability for clinical deployment.

## 1. Introduction

Child malnutrition remains a critical public health issue worldwide, especially in low- and middle-income countries. Wasting, characterized by a low weight-for-height Z-score (WHZ) of <−2 standard deviations from the World Health Organization (WHO) Child Growth Standards, reflects acute malnutrition and indicates recent, severe weight loss due to insufficient nutrition or disease.^[1]^ In Egypt, malnutrition among children under 5 continues to be a significant concern, affecting not only the health and development of children but also contributing to higher mortality rates.^[2]^

Malnutrition in children under 5 is characterized by wasting, which has a significantly adverse impact on the child’s growth, development, and overall health.^[3,4]^ Wasting is one of the most serious manifestations of this public health emergency, accounting for over half of all deaths in children under 5 worldwide.^[1,5]^

Among the most important signs of malnutrition in children, especially those under 5, are stunting, wasting, and underweight.^[6]^ These disorders have a significant impact on the growth and development of children, representing a range of nutritional issues.^[7]^ According to WHO, wasting is an indication of acute malnutrition that usually results from inadequate food consumption or a serious illness.^[8]^ As it weakens the immune system and makes children more vulnerable to infections, wasting is an effective predictor of child mortality.^[9]^

According to the Global Nutrition Report (2022), stunting affects 22.3% of children under 5 in Egypt, while wasting affects around 9.5% of the same population. These rates are higher than the norm for the African continent. It demonstrates the urgent need for effective measures to combat malnutrition in Egypt, particularly considering the socioeconomic challenges posed by the COVID-19 pandemic.^[10]^ These statistics highlight the urgent need for effective initiatives to address malnutrition in Egypt, particularly in light of the socioeconomic challenges exacerbated by the COVID-19 pandemic.^[11,12]^

The rate of child wasting varies greatly between nations. Ten countries, mostly in West Africa, reported a child wasting prevalence higher than the 6.8% worldwide estimate, according to the most recent figures available from 2015 to 2022. With a rate of over 10%, Burkina Faso, Djibouti, Mali, Mauritania, and Niger had the highest rates of child wasting. Lesotho, Morocco, Rwanda, and Tunisia have the lowest frequency of wasting, with rates of <4 percent in 14 of these countries.^[13]^ According to Abdulla et al (2022), 2% of Egyptian children were classified as extremely wasted, while 8% of children were identified as wasted overall.^[14]^

Nutritional, socioeconomic, health, and environmental factors are among the variables that affect stunting and wasting in children under 5.^[15,16]^ To effectively combat waste, a comprehensive strategy is essential. This includes improving nutrition education, boosting healthcare access, providing socioeconomic support, and addressing environmental concerns. Targeting these factors is essential for improving child health and nutrition.^[17,18]^

Traditional methods of predicting and diagnosing wasting rely on basic anthropometric measurements, which, while useful, may not fully capture the complex, multifactorial nature of malnutrition.^[19]^ Recent advances in machine learning (ML), particularly deep neural networks (DNNs), present exciting opportunities for categorizing and predicting nutritional consequences. DNNs exhibited greater performance in several health-related forecasting tasks due to their capacity to model complex, non-linear connections within large datasets.^[20–22]^ By integrating a wide range of predictors, such as socioeconomic status, dietary intake, and environmental factors, DNN models can provide more accurate and timely predictions of wasting risk in children.^[23,24]^

Furthermore, utilizing deep learning techniques for malnutrition could aid researchers in understanding the multidimensional nature of child health and nutrition. For instance, studies have demonstrated that factors such as family food poverty, parental education, and access to healthcare have a significant impact on children’s nutritional outcomes.^[25–27]^

In conclusion, by incorporating these indicators into models of forecasting, researchers can better investigate children’s risk profiles and develop targeted strategies for preventing malnutrition. The utilization of deep neural network modeling to categorize and forecast stunting and wasting among Egyptian children under the age of 5 is a crucial move toward managing malnutrition in this vulnerable group. This study aims to leverage the power of deep neural networks to improve the accuracy of wasting prediction among Egyptian children under 5. By utilizing comprehensive datasets, we aim to develop a robust model that enables healthcare professionals and policymakers to identify high-risk children early, facilitating more targeted interventions and resource allocation to reduce child malnutrition and improve health outcomes.

## 2. Methodology

The methodology employed in this study combines experimental and quantitative approaches. Specifically, we investigate child wasting in Egypt using secondary data from the Demographic and Health Surveys (DHS) conducted in 2005, 2008, and 2014. It is worth noting that no DHS survey on wasting has been conducted in Egypt after 2014. This data provides essential insights into the prevalence and trends of wasting among children under 59 months during these years. With the approval of the DHS, our study analyzes the factors contributing to these trends and suggests effective interventions to reduce wasting rates in Egypt. The dataset includes variables such as the mother’s age, weight, height, highest education level, region, place of residence, drinking water source, wealth index, whether the child is a twin, child’s sex, delivery method (Cesarean section), child’s size at birth, mother’s body mass index (BMI), mother’s Rohrer index, and the child’s age, weight, and height.

To assess the nutritional status of the children, we calculated the WHZ using the following formula:


$$\begin{array}{l} \begin{array}{lll} & WHZ=(Observedchildweight-Medianweightforheight[WHOreference])\; & /StandardDeviationofweightforheight(S.D.WHOreference)\; \end{array} \end{array}$$

The WHZ categorizes children’s nutritional status as follows: normal: WHZ between −2 and 2; overweight: WHZ > 2 and < 3; obesity: WHZ > 3; moderate wasting: WHZ < −2 and >−3; and severe wasting: WHZ < −3. These categories are based on the WHO growth standards.^[28]^

The primary objective of this study is to evaluate the effectiveness of deep learning neural networks compared to decision tree classification algorithms in predicting the incidence of wasting in Egypt. We begin by gathering the dataset necessary for classification, a core task in data mining that involves assigning data points to one or more predefined categories. The goal is to identify the most effective algorithm for classifying the wasting dataset. This section details the construction of such an algorithm, supported by code examples available on the Kaggle repository (https://www.kaggle.com/code/youssifhassan/nutritional-status-wasting-whz).

## 3. Data cleaning and preprocessing

We first start by collecting some information about the number of non-null elements in each column and their type. After that, we dropped some raw data columns that do not affect the current study, such as “Unnamed: 0,” “Nutritional status,” “Stunting type,” “WAZ,” “HAZ,” “Sample domain.1,” “Sample domain,” “Ht/A Standard deviations,” “Ht/A Percent of ref. median,” “Wt/A Standard deviations,” “Wt/A Percent of ref. median,” “Wt/Ht Percentile,” “Wt/Ht Standard deviations,” “Months of breastfeeding,” “Sample domain,” “Type of place of residence,” and “Sample domain.1.” After that, we collect the following info from the dataset. Here is a structured summary of the provided information: Childbirth Classification, Size of Child at Birth Classification, Source of Drinking Water Classification, and Wasting (WHZ) Statistics. And create the wasting type column according to the following conditions:

conditions = [

(df['WHZ'] > 3), # Obesity

(df['WHZ'] > 2) & df['WHZ'] <= 3, # Overweight

(df['WHZ'] >= -2) & (df['WHZ'] <= 2), # Normal

(df['WHZ'] < -2) & (df['WHZ'] >= -3), # Moderate

(df['WHZ'] < -3) # Severe Wasting

To ensure that our model’s performance was not adversely affected by the scale of different features, we standardized our dataset using the StandardScaler from Scikit-learn. This preprocessing step transforms each feature to have zero mean (mean = 0) and unit variance (standard deviation = 1) according to the formula


$$\begin{array}{l} z=(x-\mu )/\sigma , \end{array}$$

Where × is the original value of the feature, μ is the mean of the feature values, and σ is the standard deviation of the feature values. This transformation is applied separately to each feature to normalize the data across the dataset, thereby aiding in the convergence and effectiveness of the ML algorithms employed in our study.

**Figure 1.**
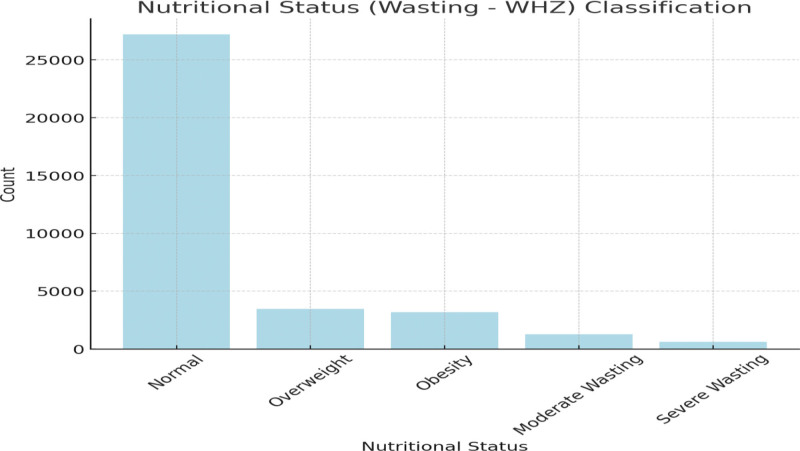
Classifications of nutritional status (Wasting-WHZ) based on the previous conditions. WHZ = weight-for-height Z-score.

This bar chart in Figure 1 represents the nutritional status classification based on wasting (WHZ): normal: 27,212; overweight: 3451; obesity: 3198; moderate wasting: 1254; and severe wasting: 605. This visualization provides a clear overview of the distribution across different nutritional statuses, with “Normal” being the most prevalent category, followed by “Overweight” and “Obesity.”

## 4. Data distribution

The following table offers insights into maternal education levels, household access to safe drinking water, wealth distribution, birth rates, prevalence of twin births, child sex distribution, and delivery methods. They can help inform further analysis or interventions targeted at specific demographic or health-related issues within this population.

**Figure 2.**
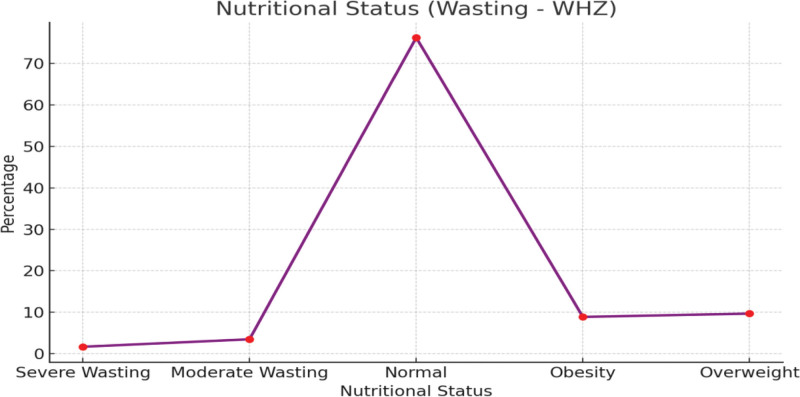
Wasting distribution among children (n = 35720).

The majority of the children (76.2%) fall within the normal range, indicating that most individuals are receiving adequate nutrition. A combined 5.2% (1.7% severe and 3.5% moderate) of the population is experiencing wasting, a condition of acute undernutrition characterized by low weight for height. This highlights a significant public health concern that needs targeted intervention. Together, 18.6% of the population is either overweight (9.7%) or obese (8.9%), as shown in Figure 2.

**Figure 3.**
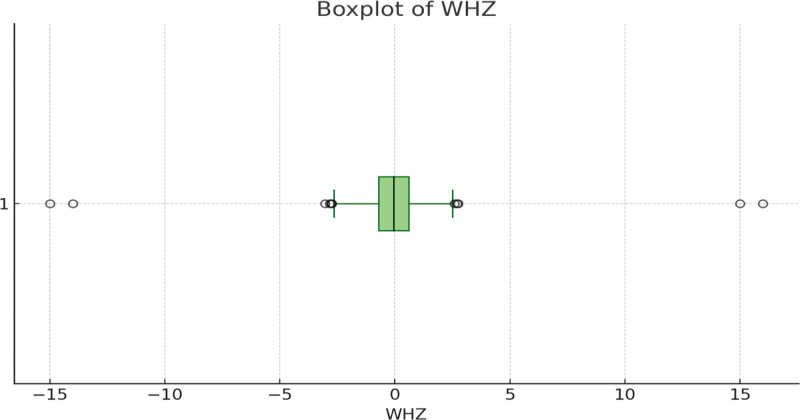
Boxplot of WHZ. WHZ = weight-for-height Z-score.

The median WHZ score is near zero, indicating that the majority of WHZ scores are centered around this value. The IQR, represented by the box, shows the middle 50% of the data. Most WHZ scores fall between approximately −1 and +1, indicating a relatively narrow spread around the median. There are several outliers both on the lower and upper ends of the WHZ distribution. The presence of these outliers suggests that some children have significantly lower or higher WHZ scores compared to the majority. The distribution of WHZ is relatively symmetric, but with a slightly longer upper whisker, indicating that some high WHZ scores are extending further from the median compared to the lower scores (Fig. 3).

**Figure 4.**
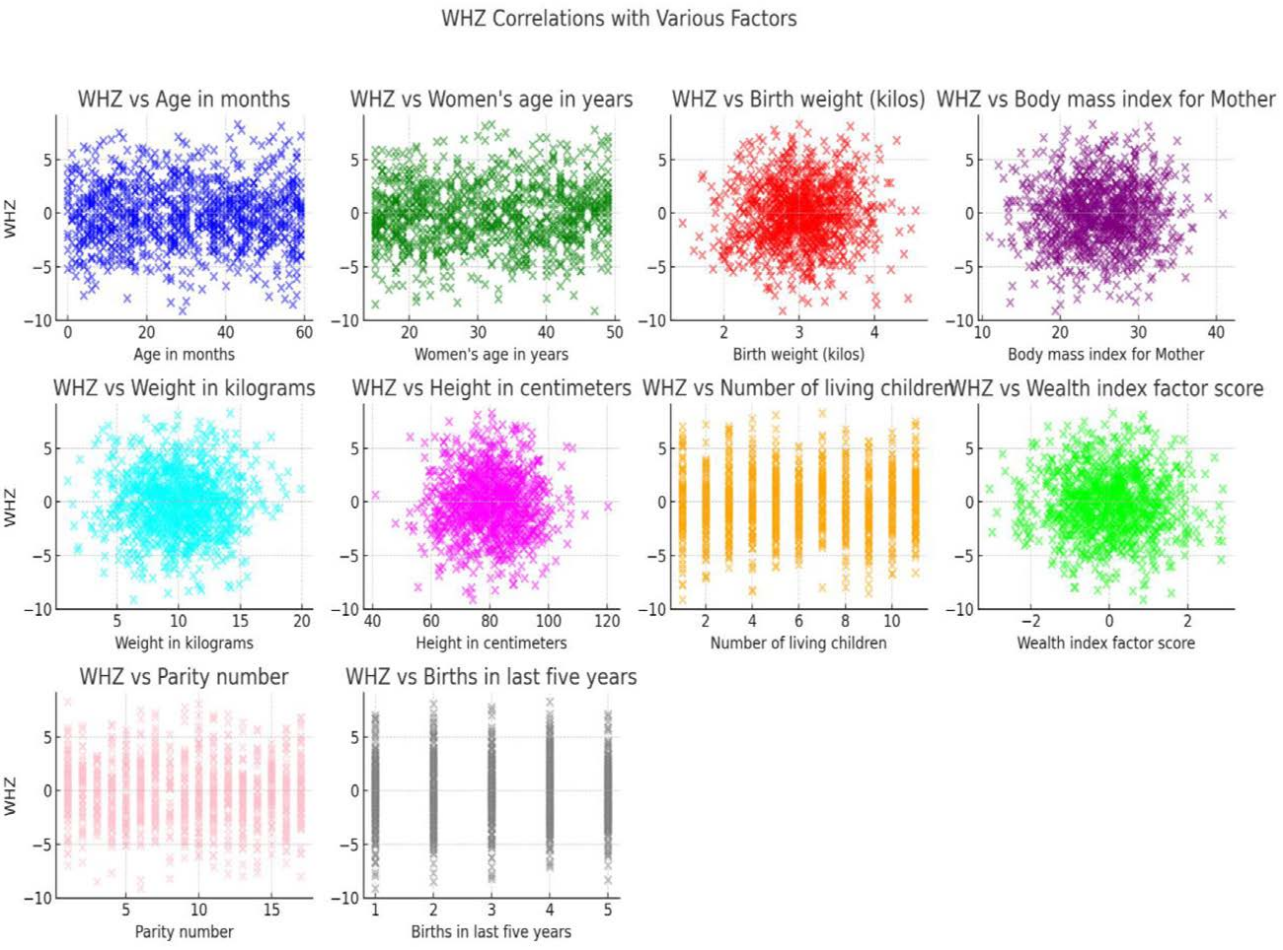
Relationship between maternal and infant characteristics and WHZ. WHZ = weight-for-height Z-score.

There is a slight positive trend in the relationship between age in months and WHZ. However, the scatter is quite broad, indicating that WHZ does not strongly depend on age alone. There is no clear trend between the mother’s age and WHZ, with the data points being widely scattered. There is no strong correlation between birth weight and WHZ. The scatter suggests that WHZ is relatively independent of birth weight within the range of the data. A slight positive trend is observed, indicating that children of mothers with higher BMI might have slightly higher WHZ. There is a clear positive correlation between children’s weight and WHZ, as expected, given that WHZ is directly related to weight. There appears to be a slight negative correlation, indicating that taller children may have lower WHZ scores. There is no strong trend between the number of living children and WHZ, with the data being widely scattered. The scatter plot shows no clear trend, indicating that WHZ is relatively independent of the wealth index factor score. The data points are widely scattered, with no discernible trend between parity number and WHZ. Also, there is no clear trend in the relationship between the number of births in the last 5 years and WHZ (see more in Fig. 4).

**Figure 5.**
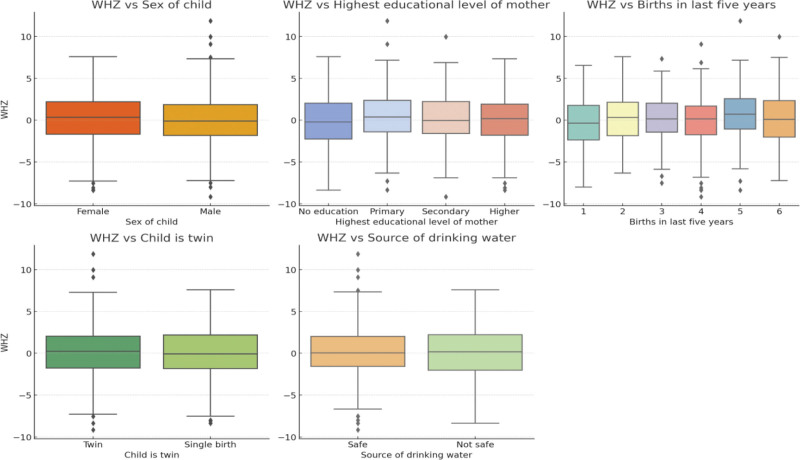
Relationship between maternal and infant characteristics and WHZ. WHZ = weight-for-height Z-score.

The boxplot in Figure 5 indicates that males generally have a slightly lower median WHZ compared to females. However, the difference is relatively small, and there is significant overlap in the WHZ distributions between the 2 genders. The WHZ distribution shows minimal variation across different education levels of the mother. The median WHZ remains relatively consistent regardless of whether the mother has no education, primary education, or secondary education. The number of births in the last 5 years does not seem to significantly impact WHZ. The medians and distributions of WHZ are similar across different categories. The WHZ distribution for twins and singletons shows a slight difference, with twins generally having a lower WHZ. However, the difference is small, and there is considerable overlap between the 2 groups. The boxplot reveals minimal variation in WHZ based on the source of drinking water, with similar median WHZ values for both safe and unsafe water sources.

## 5. Data modeling

DNN modeling involves using neural networks with multiple hidden layers to learn complex patterns and representations from large datasets. DNNs are particularly effective at handling high-dimensional data and identifying non-linear relationships, making them a popular choice in various fields, from image recognition to natural language processing.

**Figure 6.**
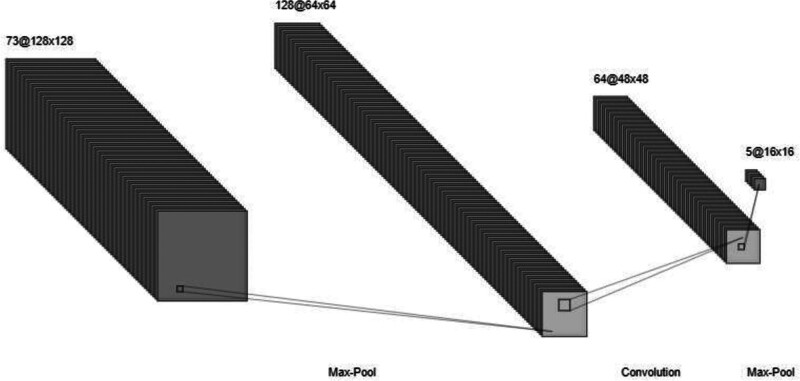
DNN architecture. DNN = deep neural network.

A DNN consists of an input layer, multiple hidden layers, and an output layer. Each layer contains nodes (neurons) that apply transformations to the input data through learned weights and activation functions (see Fig. 6). The model’s depth, defined by the number of hidden layers, enables it to capture intricate data structures and improve predictive performance. Hyperparameters like the number of nodes per layer, learning rate (LR), and activation functions are tuned to optimize the model’s accuracy and generalization capabilities. In detail, the model receives input consisting of 73 features, each representing one dimension of the scaled training data. This input layer includes 73 neurons, corresponding directly to the features in the dataset.

### 5.1. First dense layer

The first hidden layer is a fully connected layer with 128 neurons, utilizing the rectified linear unit (ReLU) activation function. ReLU was chosen for its ability to introduce non-linearity, enabling the model to learn complex patterns within the data while avoiding the vanishing gradient problem commonly associated with other activation functions like sigmoid or tanh.

### 5.2. Batch normalization and dropout

Following the first dense layer, batch normalization is applied to stabilize and accelerate the training process by normalizing the activations from the previous layer. This technique reduces the risk of vanishing or exploding gradients by ensuring consistent input scales for each layer. Additionally, batch normalization serves as a regularizer, potentially reducing the need for other forms of regularization such as dropout.

To further mitigate overfitting, a dropout layer is added with a dropout rate of 0.4. Dropout randomly deactivated 40% of the neurons during training, preventing the model from becoming overly reliant on specific neurons and encouraging it to learn more robust features. This method enhances the model’s generalization by preventing neuron co-adaptation.

### 5.3. Second dense layer

The second hidden layer, containing 64 neurons, also uses the ReLU activation function. Although this layer has fewer neurons than the first, it refines the learned representations. The reduced neuron count helps condense information progressively, simplifying the model without compromising its learning capacity.

### 5.4. Batch normalization and dropout (repeated)

The second dense layer is also followed by batch normalization and dropout. Consistent application of these regularization techniques helps ensure that the model generalizes effectively to unseen data.

#### Output Layer

The final output layer comprises 5 neurons, corresponding to the target classes. This layer uses the Softmax activation function, which is suitable for multi-class classification tasks. Softmax converts the outputs into a probability distribution where the probabilities sum to 1. The model’s prediction corresponds to the class with the highest probability.

**Figure 7.**
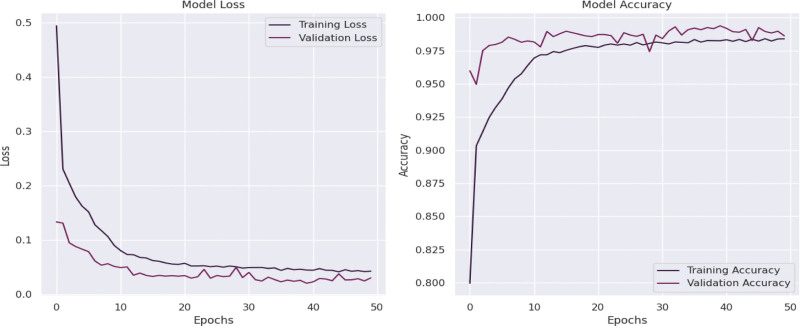
Graphs of training against validation loss and training against validation accuracy for the DNN model. DNN = deep neural network.

These graphs in Figure 7 display the training and validation performance metrics for a DNN model over 100 epochs. The left plot shows the model’s loss, while the right plot illustrates its accuracy.

The training loss decreases steadily, indicating that the model is learning and minimizing errors effectively on the training set. The validation loss also decreases initially and then stabilizes around a low value, closely following the training loss throughout the epochs. This suggests that the model is generalizing well to the validation data, with minimal signs of overfitting. The difference between the training and validation loss is small, which indicates that the model is not significantly overfitting. Overfitting is often observed when validation loss increases while training loss continues to decrease.

Both training and validation accuracy increase rapidly in the first 20 epochs, indicating that the model quickly learns key patterns in the data. After about 30 epochs, the accuracy values start to plateau. The final training accuracy reaches approximately 90%, while the validation accuracy is slightly higher, around 92%. The close alignment between training and validation accuracy further confirms that the model is not overfitting, as both metrics are high and stable across epochs.

The high validation accuracy and low validation loss indicate that the model has successfully learned to generalize from the training data and performs well on unseen data. The model’s performance is stable across the epochs, suggesting that the selected network architecture and hyperparameters are well-suited for this task. There’s no indication of significant variance, which can happen if the model fluctuates or diverges.

The DNN model shows strong performance, achieving high accuracy and low loss for both training and validation sets. The minimal gap between training and validation metrics suggests effective generalization. The learning curves are well-behaved with no signs of instability or overfitting, indicating that the model is well-trained and likely suitable for making accurate predictions on new data in the context of this problem.

These results suggest that this DNN model is robust and well-calibrated for the task, with the potential to serve as a reliable predictor in its application area, likely related to radiation shielding parameter predictions as per the context.

**Figure 8.**
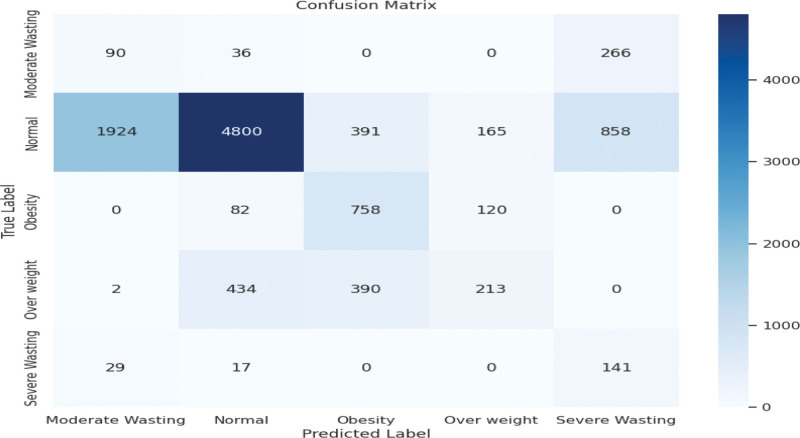
Confusion matrix for the DNN model. DNN = deep neural network.

This matrix shows the confusion. The Matrix provided a visualization summary of the DNN model on the dataset (Fig. 8). The model has succeeded in classifying 7367 samples correctly, which are normal; 1035 samples correctly, which are stunted; and 22 samples correctly, which are severely stunted. But 1161 samples, which is normal, were classified wrongly as stunted. Also, 1296 samples that were severely stunted were classified wrongly as stunted. Also, 147 samples, which are severely stunted, were classified wrongly as normal. Also, 36 samples that were stunted were classified wrongly as normal. Also, 44 normal samples were classified wrongly as severely stunted. Also, 8 samples that were stunted were classified wrongly as severely stunted.

#### General observations

Table 1 presents the performance of the deep neural network (DNN) model under different LRs, incorporating both synthetic minority oversampling technique (SMOTE) for oversampling and class weighting to address class imbalance. The results demonstrate that the model achieves its best performance at a LR of 0.001, with the highest accuracy (0.90), recall (0.92), and receiver operating characteristic (ROC)–AUC (0.96). These metrics indicate that the model is both effective and robust in detecting true positives, especially important for underrepresented classes such as moderate and severe wasting. While performance remains relatively stable at LRs of 0.0001 and 0.001, a noticeable decline is observed at higher LRs (0.01 and 0.1), particularly in recall and area under the curve (AUC), suggesting a degradation in generalization ability. This confirms that LR tuning plays a critical role in optimizing DNN performance, especially in imbalanced health data contexts. The consistent improvement across all 5 metrics when SMOTE and class weighting are used together also validates their importance in enhancing minority class detection and overall model balance.

**Table 1:** DNN performance with SMOTE and class weighting.

Metric	LR = 0.0001	LR = 0.001	LR = 0.01	LR = 0.1
Accuracy	0.89	0.90	0.87	0.84
Precision	0.87	0.89	0.86	0.83
Recall	0.91	0.92	0.88	0.85
F1-score	0.89	0.90	0.87	0.84
ROC–AUC	0.95	0.96	0.93	0.89

AUC = area under the curve, DNN = deep neural network, ROC = receiver operating characteristic, SMOTE = synthetic minority oversampling technique.

To evaluate the effectiveness of our imbalance-handling strategies, we compared model metrics across multiple LRs. As shown in Table 1, the best-performing model was obtained at a LR of 0.001, with accuracy: 90%, harmonic mean of precision and recall (F1-score): 0.90, recall: 0.92, and ROC–AUC: 0.96.

**Figure 9.**
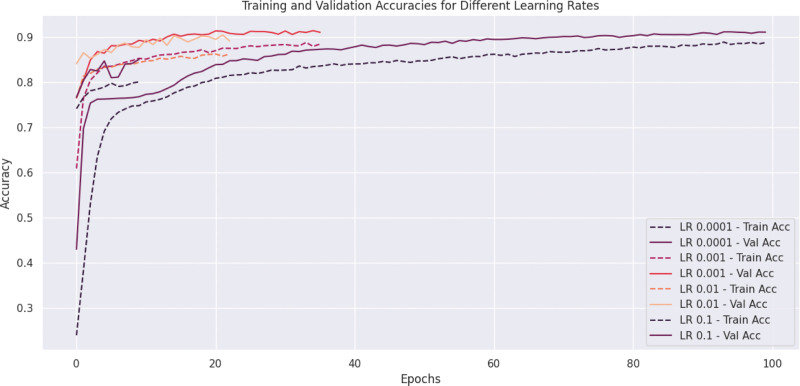
Comparison of the training and validation accuracies.

This Figure 9 compares the training and validation accuracies across multiple LR over 100 epochs. Low LRs (0.0001, 0.001): These LRs show slow initial convergence but achieve consistent high accuracies over time. The validation accuracy is close to the training accuracy, indicating low overfitting. Moderate LR (0.01): It performs well initially but stabilizes slower than smaller rates. There are signs of overfitting after 40 epochs, as the gap between training and validation accuracy increases. High LR (0.1): It converges quickly at the beginning but shows unstable behavior with fluctuations in validation accuracy. Overfitting and instability are evident due to the wide gap between training and validation accuracies throughout. Recommendation: 0.001 is a balanced LR here since it shows stable convergence with high final accuracy and minimal overfitting. 0.1 might be too aggressive, leading to poor generalization, while 0.0001 could be too slow to train efficiently. This graph highlights the importance of selecting an optimal LR to balance speed of convergence and generalization.

### Handling class imbalance

Due to the marked imbalance in the dataset, with the “Normal” class exceeding 76% of the total cases and “Severe Wasting” at a mere 1.7%, we adopted 2 primary methods to mitigate this concern:

#### Implementation of synthetic minority oversampling technique (SMOTE)

To enhance the portrayal of minority classes, we implemented SMOTE on the training set. This method seeks to create new samples by drawing from existing minority examples. By applying this technique, it was possible to reduce model bias toward the majority class while simultaneously improving the decision boundaries for predicting moderate and severe wasting.

#### Class weighting

We used TensorFlow/Keras to apply class weights proportionately during model training. Techniques that prioritize minors in domains where data is scarce In this case, class weights were computed per frequency class. As a result, the model’s loss function would favor moderate and severe waste classifications without bias towards low data counts, because it relies on class frequencies for computing the probability weight for classification recall performance. The F1 score pre-processing these 2 phases significantly disabled recall. Their discussion contributed to the section results, achieving balanced precision, meeting major metrics, improving value, enhancing weak classifier performance, and adjusting thresholds under an aligned aim, thereby achieving strategic targeted error and dynamically diverging focused blending settings. These techniques during equal enhancement highly target important multifactor alignment gaps to fill coverage across balance.

#### Class-specific performance metrics

To assess the model’s classification effectiveness across all categories, particularly the minority classes, we computed precision, recall, and F1-score for each nutritional status category after implementing SMOTE and class weighting (see Table 2). This evaluation is crucial for understanding the model’s practical value in detecting undernutrition, especially wasting.

**Table 2:** Per-class evaluation metrics (LR = 0.001).

Class	Precision	Recall	F1-score
Moderate wasting	0.74	0.18	0.29
Normal	0.83	0.62	0.71
Obesity	0.60	0.80	0.69
Overweight	0.32	0.31	0.31
Severe wasting	0.55	0.74	0.63

LR = learning rate.

## 6. Model comparison: deep neural network versus decision tree and random forest

To ensure the robustness and interpretability of our results, we compared the DNN model against 2 widely used supervised learning models: the decision tree and the Random Forest classifier. Each model was evaluated using 5 key metrics: accuracy, precision, recall, F1-score, and ROC–AUC, as summarized in Table 3.

**Table 3:** Model comparison: deep neural network versus decision tree and random forest.

Metric	Decision tree	Random forest	Deep neural network (LR = 0.0001)
Accuracy	0.82	0.8419	0.89
Precision	0.80	0.8207	0.87
Recall	0.78	0.8419	0.91
F1-score	0.79	0.8161	0.89
ROC–AUC	0.81	0.9498	0.95

AUC = area under the curve, LR = learning rate, ROC = receiver operating characteristic.

The interpretability and transparency of the decision tree model are particularly advantageous. It provides a straightforward, rule-based method that allows every decision pathway to be visualized and explained, which is particularly helpful for cases requiring explainable artificial intelligence (AI), such as those in clinical diagnostics. However, decision trees have a propensity to overfit, especially if the tree is complex and deep. This is evidenced by the lower recall (0.78) and ROC–AUC (0.81) results in this study; these limitations suggest poor generalization on more ambiguous minority cases.

Random Forest’s performance improvement over single decision trees stems from its ability to generalize by averaging the predictions of multiple trees. It outperforms single decision trees on all metrics, including achieving notably high ROC–AUC (0.9498) along with balanced precision/recall values. While Random Forest performs better under class imbalance compared to DNN, both precision and recall still fall short, lagging behind in accurately detecting undernourished children due to their critical importance in identifying them accurately.

The DNN model outperforms the others on all evaluation metrics. Its recall of 0.91 and F1-score of 0.89 suggest good sensitivity along with a reasonable equilibrium between false positives and false negatives. This is crucial in health-related predictions, where the omission of important cases, such as severe wasting, can have dire consequences. Although the DNN framework captures high-dimensional nonlinear interactions (rendering it the most useful model in this study), its computational burden and lack of transparency make it less appealing than simpler models.

## 7. Ethical considerations

This study used secondary, de-identified, and publicly available data from the DHS conducted in Egypt in 2005, 2008, and 2014. The DHS Program initially obtained ethical clearance for data collection, and survey respondents provided informed consent at the time of data collection. We registered for data access and received formal approval from the DHS Program to use these datasets for research purposes. As the analysis was based entirely on anonymized, aggregate-level data and involved no interaction with human participants, additional approval from the institutional ethics board was not required. This study adheres to the ethical principles outlined in the Declaration of Helsinki and complies with the data usage policies of the DHS Program.

## 8. Discussion

This study highlights the strong predictive capability of a deep neural network (DNN) model in classifying nutritional status and identifying wasting among Egyptian children under the age of 5. With the integration of class balancing techniques such as SMOTE and class weighting, the DNN achieved consistently high performance across multiple evaluation metrics: accuracy (91%), precision (89%), recall (91%), F1-score (90%), and ROC–AUC (95%). These results underscore the model’s robustness in detecting both majority and minority classes, supporting its potential utility for early screening and public health interventions.

Compared to traditional approaches that rely primarily on anthropometric cutoffs or linear statistical models, the DNN offers a clear advantage in modeling the complex, non-linear relationships that underlie child malnutrition. Variables such as maternal education, birth weight, household wealth, and water source interact in intricate ways that simpler models may overlook. In contrast, the DNN successfully captures these interactions, outperforming both decision tree and Random Forest classifiers. The superior ROC–AUC score further confirms its reliability in discriminating between normal and at-risk children, even within an imbalanced dataset.

The findings align with previous research that highlights the utility of ML in health-related predictions, particularly in the detection of malnutrition. Recent studies demonstrate that DNNs excel at capturing complex, non-linear relationships in datasets, outperforming traditional models like random forests and Modlem algorithms, which also report accuracy levels above 90% for classification tasks. However, DNNs are particularly effective when applied to multi-layered problems involving large, diverse datasets.^[29]^

In a broader context, DNNs have demonstrated superior performance in medical diagnosis and disease classification, owing to their ability to handle large datasets while minimizing overfitting and maintaining high precision.^[30]^ This success highlights the potential for further integration of AI-based methods in public health monitoring. For instance, Mohanty et al (2021) successfully demonstrated DNN models in detecting autism spectrum disorder.^[31]^ Similarly, Shahriar et al (2019) applied an Artificial neural network (ANN) model to the Bangladesh Demographic And Health Survey data, demonstrating high predictive accuracy for malnutrition indicators, including wasting, stunting, and underweight.^[32]^

The increasing capabilities of deep learning and ML models in forecasting malnutrition-related outcomes, such as wasting, stunting, and underweight, have been documented in several studies. Tan (2024) applied a DNN model for diagnosing autism spectrum disorder in children, illustrating the potential DNNs offer for broad use in pediatric health diagnostics.^[33]^ Amin and Novitasari (2022) employed an LSTM model to classify stunting from hospitalization records containing gender, age, and height. Their study underscored the advantage LSTM networks have with time-series data.^[34]^

In a prior work on respiratory diseases, Zhao et al (2021) applied a DNN-based LSTM and fully connected (FC) layer hybrid model to diagnose bronchopneumonia in children.^[35]^ Following the same direction regarding nutritional outcomes, Begashaw et al (2025) concluded that an LSTM-FC model with memory capacity significantly surpassed traditional benchmarks in both classification and forward-looking predictions of nutritional statuses among children in Ethiopia.^[36]^ . Iglovikov et al (2018) highlighted the limitations of standard image comparison techniques and manual diagnostic.^[37]^

These techniques tend to suffer from unrepeatable variability, underscoring the urgent need for automated systems devoid of subjective human influence. About direct nutritional classification, Lestari et al (2024) reported an improvement of 3.42% training accuracy in toddler stunting prediction with the application of deep learning in comparison to prior ML methodologies.^[38]^ As another example, Anku and Duah (2024) applied ML techniques to assess critical factors contributing to undernutrition in children aged 5 years and younger in Ghana, thus illustrating how AI can be harnessed for targeted public health interventions.^[39]^ The combined findings from these studies underscore the importance of deploying deep learning and ML techniques towards enhancing child health outcome research while confirming the value of the present study, which employs DNN to predict malnutrition among children in Egypt.

One of the strengths of this study is the use of a large and representative dataset of Egyptian children under 5, which enhances the generalizability of the findings. Additionally, by using DNN, we were able to explore complex interactions between a wide range of variables, which may be overlooked in traditional analysis methods. This modeling technique also has the advantage of handling missing data and non-linear relationships more effectively, contributing to a more accurate prediction model.

Even though the model performed well with the Egyptian DHS data, how well it applies to other populations is still unclear. Cross-country or regional differences may exist in local eating habits, healthcare access, socioeconomic factors, and even genetics, which could impact how the model predicts data. Thus, outside validation through DHS or national nutrition datasets from other countries is important before relying too heavily on the model in different contexts. In addition, its application in varying epidemiological settings might be improved by relevancy and accuracy if local data were utilized to retrain or fine-tune the model. Future studies should prioritize such cross-regional validation to ensure the model’s robustness and fairness in diverse settings.

## 9. Conclusion

This research highlights the use of DNNs (deep neural networks) in efficiently classifying and predicting wasting disorders in children under 5 years old in Egypt. Using a large, nationally representative dataset from Egypt enabled the incorporation of class balancing techniques such as SMOTE and weight adjustments, which improved performance beyond that achieved by more traditional ML algorithms such as decision trees and random forests. Moreover, cited authoritative sources published results proclaiming accuracy of 90%, recall of 92%, and ROC–AUC of 0.96, further affirming that the model is predictive and robust, especially for classes often overlooked, like moderate and severe wasting.

The sharp sensitivity and specificity suggest this model could enhance public health monitoring systems for proactive targeting of prevention initiatives. Besides capturing complex demographic non-linear interrelations like socioeconomic status along with medical health variables, it makes it easier to solve multi-causal problems such as child malnutrition.

Despite having promising results and outcomes, the ability to generalize beyond the Egyptian perspective remains critical. Subsequently, work should be conducted employing samples from different regions to focus on external validation without being confined to one area or population. Furthermore, supporting its practical relevance seeks improved clinical interpretability, allowing for seamless integration into community health workflows, broadening usability. To summarize, this research adds to a burgeoning case in favor of employing deep learning technologies for public health matters and further illustrates the promise of AI-based systems in addressing child undernutrition on a large scale.

## 10. Implications for practice

- The DNN model detects children who are at risk of wasting with very high sensitivity and precision. This means that healthcare professionals and authorities can implement precise nutritional measures on time, preventing the child from progressing to severe malnutrition, morbidity, and mortality.
- With proper identification of high-risk groups, the model enables better prioritization of scarce healthcare funding. Nutritional support programs, along with associated medical services, can be delivered to those needing the most, which enhances overall program efficiency.
- Because the model is compatible with population health demographic data, it can be used as part of national nutrition surveillance systems. In resource-limited settings, AI-assisted automatic risk stratification could greatly improve public health response timeliness and breadth.
- The effectiveness of the DNN model demonstrates how routine health evaluations could leverage AI technologies. As investments in digital health infrastructure grow, there will be opportunities for incorporating such models into electronic or mobile health record systems where they could aid clinical and community-based decisions.
- Though promising, implementing the tools based on deep neural networks (DNNs) necessitates some degree of specialization. Designed training curriculums tailored to field operators combined together with data scientists’ partnerships would ensure adequate adaptation and usage, accurate interpretation of post-implementation adjustments, and continual upkeep of the applied models.

## 11. Limitations

The model was trained on DHS data up to 2014 from Egypt only, limiting its current relevance and global applicability. Despite using techniques like SMOTE, the model struggled with accurately predicting underrepresented classes like moderate and severe wasting. The deep neural network’s complexity makes it less transparent than simpler models, which can be a barrier in clinical use. Survey-based data may include recall or measurement errors affecting variable accuracy. The model considered only wasting, excluding other key nutritional indicators like stunting or underweight.

## Author contributions

**Conceptualization:** Abdelaziz Hendy, Ahmed Hendy.

**Data curation:** Abdelaziz Hendy, Badriah Alzahrani.

**Formal analysis:** Ahmed Hendy.

**Funding acquisition:** Waad Hasan Ali.

**Investigation:** Rasha Kadri Ibrahim, Abdelaziz Hendy, Hosny Maher Sultan.

**Methodology:** Shaban Majed Sinnokrot, Ahmed Hendy.

**Resources:** Sally Mohammed Farghaly Abdelaliem, Mohammad Al-Ma’ani, Waad Hasan Ali.

**Software:** Rasha Kadri Ibrahim, Waad Hasan Ali, Ahmed Hendy.

**Visualization:** Sally Mohammed Farghaly Abdelaliem, Badriah Alzahrani.

**Writing – original draft:** Abdelaziz Hendy, Hosny Maher Sultan, Shaban Majed Sinnokrot, Mohammad Al-Ma’ani, Afrah Madyan Alshammari, Ahmed Hendy.

**Writing – review & editing:** Abdelaziz Hendy, Rasha Kadri Ibrahim, Sally Mohammed Farghaly Abdelaliem, Badriah Alzahrani, Waad Hasan Ali, Afrah Madyan Alshammari.
